# Responses to serious adverse incidents in mental health care settings: a qualitative study of a complex patient safety system

**DOI:** 10.1192/j.eurpsy.2024.1270

**Published:** 2024-08-27

**Authors:** N. Anderson, R. Nathan

**Affiliations:** Cheshire and Wirral Partnership NHS Foundation Trust, Chester, United Kingdom

## Abstract

**Introduction:**

Individual patient safety processes (such as reporting, investigating, learning and improving patient outcomes) activated following serious adverse incidents (e.g. patient suicide) are not distinct or standalone. Rather, they are embedded within a complex system of multiple interdependent processes enacted by individuals who are subject to an array of implicit and explicit influences (Nathan *et al.* BJPsych Advances 2022; 1-11). Although some specific elements of the response to adverse incidents have been examined, no previous empirical research has set out to study the complex interacting system within which these elements are situated.

**Objectives:**

This study’s aim was to characterise a complex patient safety system and to identify types of processes across that system that have an impact on the goal of improving patient safety.

**Methods:**

Recorded 1:1 semi-structured interviews were undertaken with staff in a range of patient safety roles across a mental health care system to elicit accounts of the system response to serious adverse incidents. These interviews were transcribed, and the transcriptions were subject to thematic analysis using the *Framework Method* for qualitative research in health care settings (Gale *et al.* BMC Med. Res. Methodol. 2013; 13.1; 1-8).This preliminary study relates to the analysis of 8 interviews.

**Results:**

The following six main types of influences on the effectiveness of patient safety system responses to adverse incidents were identified:

1. **Differing functions/expectations of investigations into serious incidents** (due to differing demands of different parties, such as the health provider, the family, the coroner, etc);
2. **Differing methodologies used to investigate serious adverse incidents** (although system-based generally preferred, there was a noted risk that this approach may fail to identify occasional examples of poor practice);
3. **Relationship between incident investigation processes and patient safety processes** (with a particular potential for the latter to dominate the system at the expense of the former);
4. **System complexity** (multiple interacting processes/processors at multiple levels within the health provider and wider health system);
5. **Operationalising recommendations from investigations** (with the potential for adverse unintended patient safety consequences)
6. **Influence of national directives**

**Conclusions:**

As well as paying attention to individual components of the safety system (e.g. investigation methodology and organisational culture), the development of an effective patient safety system is dependent on an understanding of the complex interacting processes across the system. This study sheds empirical light on key influences that act across a mental health provider system. Both researchers of patient safety and providers intending to improve their approach to patient safety should take account of such systemic influences on effectiveness.

**Disclosure of Interest:**

None Declared

